# Impact of outpatient gastroenterology consult on pharmacotherapy and management of gastrointestinal symptoms in Parkinson’s Disease

**DOI:** 10.1016/j.prdoa.2023.100215

**Published:** 2023-08-29

**Authors:** Jocelyn J. Chang, Sanjay R.V. Gadi, Aleksandar Videnovic, Braden Kuo, Trisha S. Pasricha

**Affiliations:** a Tufts University School of Medicine, Boston, MA, United States; b Department of Medicine, Duke University Health System, Durham, NC, United States; c Neurological Clinical Research Institute, Department of Neurology, Massachusetts General Hospital, Boston, MA, United States; d Harvard Medical School, Boston, MA, United States; e Center for Neurointestinal Health, Division of Gastroenterology, Department of Medicine, Massachusetts General Hospital, Boston, MA, United States

**Keywords:** Gastric dysmotility, Parkinson’s Disease, GI consultation, Motor fluctuations, Levodopa malabsorption

## Abstract

**Background & aims:**

Gastrointestinal (GI) symptoms are common in Parkinson’s Disease (PD) patients, and GI dysmotility is thought to induce motor fluctuations, requiring escalation of levodopa therapy. The role of GI consultation in managing such symptoms, however, is unclear. In this study, we investigate the possible association between GI dysmotility symptoms and escalated LEDD therapy, as well as factors associated with GI consultation for PD symptom management.

**Methods:**

This was a retrospective case-study of 248 PD patients evaluated by outpatient neurology at Massachusetts General Brigham Healthcare from 2018 to 2022. Logistic regression, *t*-test, and Fisher exact tests were performed to identify factors associated with GI consult, change in LEDD with consult, and association of consultation with GI diagnoses and treatments, respectively.

**Results:**

Among 248 PD patients, 12.9% received GI consultation despite 96.8% having GI symptoms. Bloating was the primary symptom associated with receiving GI consultation (OR 3.59 [95% CI 1.47–8.88], *p* = 0.005). GI consultation increased the odds of receiving GI-specific medications (78.2% vs 46.3%, *p* = 0.001) and specialized GI diagnoses like gastroparesis (9.4% vs 0.46%, *p* < 0.001) and pelvic floor dysfunction (15.6% vs 0%, *p* < 0.0001). Interestingly, LEDD tended not to change after GI consultation, and dysmotility symptoms, including bloating, did not predict need for higher LEDD.

**Conclusions:**

While treating symptoms of dysmotility may not ameliorate levodopa-based motor fluctuations as much as previously thought, GI consultations are underutilized in PD, and patients who receive GI consultation are more likely to have changes in GI diagnosis and treatment.

## 1 Introduction

Parkinson’s Disease (PD) is a complex, multisystem neurodegenerative disorder characterized primarily by the motor symptoms of tremors, rigidity, and bradykinesia, as well as the non-motor symptoms of cognitive and autonomic dysfunction, including gastrointestinal (GI) distress. GI dysfunction has been reported in 54.5–95% of PD patients [1], as well as in the prodromal stage before motor symptom onset [2]. However, the role of GI consultation in managing such symptoms is unclear. Over the counter (OTC) availability and non-GI specialist prescription of GI medications are common, and the benefits of GI referral in PD patients have yet to be clearly defined.

In addition to symptom management, a common reason for GI referral in PD is a concern for levodopa-based motor complications [3], [4], which has been found to occur in nearly 60% of patients after 10 years of levodopa therapy [5], and approximately 40% after 4–6 years of levodopa therapy [6], [7]. After several years of successful levodopa therapy, motor symptom control that characterized initial treatment begins to wear off sooner and with less predictability. In this scenario, levodopa equivalent daily dose (LEDD) is often increased. One common proposed etiology of these motor fluctuations is gastric dysmotility-induced levodopa malabsorption [8], [9], [10], [11].

GI motility disorders like small intestinal bacterial overgrowth (SIBO) and gastroparesis (delayed gastric emptying), with prevalence rates of 46% and 45% in the PD population, respectively [12], [13], are thought to impair levodopa absorption. Delayed gastric emptying prolongs the time for ingested levodopa to reach its absorptive sites in the proximal small intestine, resulting in delayed or even absent onset of benefit. Moreover, when retained in the stomach, levodopa can be converted to dopamine by dopa decarboxylase in the gastric mucosa, rendering it unavailable for delivery to the brain [10]. Nevertheless, existing research is still mixed concerning the effects of gastric dysmotility, if any, on levodopa pharmacokinetics [14], [15], and successful clinical strategies for addressing dysmotility-induced malabsorption, if warranted, remain elusive [7], [16].

In this study, we investigate the possible association between GI dysmotility symptoms and escalated LEDD therapy for maintaining patient motor scores, as well as factors associated with GI consultation for improved PD symptom management. Dysmotility-induced levodopa malabsorption would predict higher average motor-score adjusted LEDD values in patients with greater GI dysmotility as a result of dose escalation.

## 2 Methods

### 2.1 Study population

We identified a retrospective sample of 248 PD patients who had been evaluated at Massachusetts General Brigham (MGB) between January 2018 and August 2022.

Patients were selected using the Research Patient Data Registry (RPDR) [17], a Partners Healthcare electronic database that stores data on patient demographics, encounters, medications, procedures, and billing codes. Using the RPDR search query tool, we identified patients meeting the following inclusion criteria: (1) within the MGB system, (2) adults 18 years and older, (3) diagnosis of idiopathic Parkinson’s disease (ICD10:G20), and [4] received care from MGB outpatient neurology between 2018 and 2022. The RPDR search query was then filtered for charts containing “UPDRS'' (Unified Parkinson’s Disease Rating Scale) [18] in order to refine our cohort to patients being actively assessed for PD severity, treatment, and progression. Excluded from the cohort were patients with secondary Parkinsonism, Parkinson plus syndromes, and drug-induced Parkinson’s.

### 2.2 Data collection

Data was collected by manual chart review by study staff for: (1) PD-related patient data including PD diagnosis date, PD medications, and UPDRS for symptom severity; (2) GI-related data including GI medications, GI-specific diagnoses [Supplementary Table 1], presence of 6 GI symptoms associated with dysmotility (dysphagia, nausea, vomiting, epigastric pain, bloating, and constipation) [19], [20], history of GI consultation; and anorectal manometry testing confirming a diagnosis of pelvic floor dyssynergia [3] patient demographics and other disease comorbidities, the latter of which was converted to a numerical score using the Charlson Comorbidity Index (CCI) [21].

To improve comparability, PD medications and dosing were converted to LEDD [22]. If patients presenting with GI symptoms were not prescribed accompanying GI medications, a full search of their charts for possible OTC medication use was performed and documented. Most charts recorded patient motor scores using the UPDRS scoring system. Motor scores reported as MDS-UPDRS (the updated UPDRS) were converted to UDPRS using a Hoehn Yahr scale-based conversion for better comparability [23].

### 2.3 Statistical analyses

Based on patients’ history of GI consult, descriptive characteristics of patients were reported as means and standard deviations for continuous variables and total number and percent of total number for categorical variables. Descriptive data was then analyzed using t-tests and chi-square tests, respectively, with p-value ≤ 0.05 as statistically significant.

Logistic regression analysis was applied to evaluate: (1) factors associated with GI consultation and (2) the association of dysmotility symptoms with LEDD, both reported as odds ratios (OR) with 95% confidence intervals (CI). Covariates for the latter were selected *a priori* on the basis of possible associations with GI symptoms. Selected covariates included age, sex, race, ethnicity, CCI, and UPDRS motor scores. For PD patients with a history of GI consult, the average change in LEDD before and within 5 years after consultation was analyzed by *t*-test and presented as a bar graph, with error bars for standard deviation.

Associations of GI consultation with GI medication prescription, and GI-specific diagnoses were calculated using Fisher’s exact test. Nausea/heartburn and laxative medications were individually analyzed for association with GI consultation, as these categories of medications were by far the most commonly prescribed in our patient study.

Study analyses were performed in R (version 4.0.3), and Prism (version 8). Statistical tests were 2-sided using an alpha level of 0.05.

## 3 Results

In our cohort, no significant demographic differences were noted between patients who did (32/248) and who did not receive a GI consultation [Table 1]. Patients also had similar CCIs, average LEDD, and UDPRS 3 scores regardless of GI consultation. Regarding gastrointestinal symptoms, in a univariate screen, patients who received a GI consultation were more likely to have vomiting (p = 0.003), epigastric pain (p = 0.025), and/or bloating (p < 0.001).

**Table 1:** Demographics and characteristics of Parkinson's patients without and without GI consult between 2018 and 2022.

	No GI consult	GI consult	*p* Value
**Demographics**	**N = 216**	**N = 32**	
**Age (mean, SD)**	71.84 (9.26)	72.06 (8.51)	0.897
**Race (n, %)**			0.522
*White*	187 (86.6)	28 (87.5)	0.226
*Black*	13 (6.0)	1 (3.1)	
*Asian*	7 (3.2)	3 (9.4)	
*Other*	9 (4.2)	0 (0.0	
**Ethnicity (n, %)**			0.129
*Non-Hispanic*	173 (80.1)	29 (90.6)	
*Hispanic*	41 (19.0)	2 (6.2)	
*Other*	2 (0.9)	1 (3.1)	
**Male (n, %)**	118 (54.6)	20 (62.5)	0.518
**Charlson Co-morbidity index (mean, SD)**	3.65 (1.85)	4.22 (1.91)	0.109
**LEDD (mg) (mean, SD)**	890.57 (594.26)	883.44 (398.56)	0.948
**UPDRS 3 (off meds)**	21.22 (12.05)	22.73 (10.41)	0.55
**GI symptom (n, %)**			
*Dysphagia*	100 (46.3)	21 (65.6)	0.064
*Nausea*	112 (51.9)	23 (71.9)	0.053
*Vomiting*	50 (32.1)	16 (50.0)	**0.003**
*Epigastric Pain*	45 (20.8)	13 (40.6)	**0.025**
*Bloating*	45 (20.8)	18 (56.2)	**<0.001**
*Constipation*	179 (82.9)	31 (96.9)	0.074

From highest to lowest, the prevalence of individual GI symptoms among all patients was: 84.7% (210 of 248) for constipation, 54.4% (135 of 248) for nausea, 48.8% (121 of 248) for dysphagia, 26.6% (66 of 248) for vomiting, 25.4% (63 of 248) for bloating, and 23.4% (58 of 248) for epigastric pain. Overall, the prevalence of patients presenting with at least one of six GI symptoms was very high at 96.8% (240 of 248); however, in spite of the high prevalence, only 12.9% (32 of 248) of patients received GI consultation for their symptoms [Table 1].

**Fig. 1:**
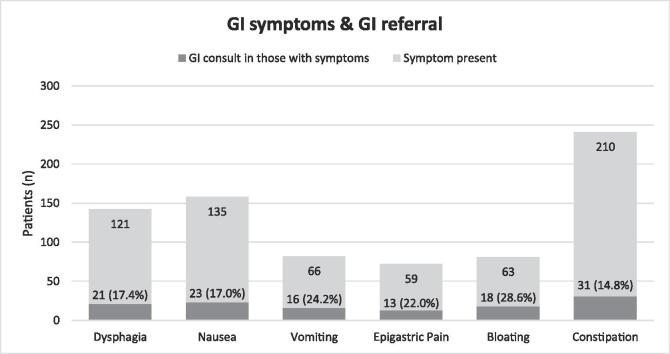
Distribution of GI Symptoms and GI Consult.

After controlling for confounders, bloating was the primary symptom associated with receiving GI consultation (OR 3.59 [95% CI 1.47–8.88], p = 0.005) [Table 2], with 28.6% (18) of total patients with bloating (63) receiving consult [Fig. 1].

**Table 2:** Logistic regression model identifying factors associated with GI referral among Parkinson's Patients.

Variable	Odds ratio (95% CI)	*p value*
**Age**	0.99 (0–8.3)	0.64
**Sex (male)**	1.69 (0.68–4.46)	0.27
**Race**		
*White*	reference	–
*Black*	0 (0–6.21)	0.98
*Asian*	4.03 (0.65–22.8)	0.11
**Ethnicity**		
*Non-hispanic*	reference	–
*Hispanic*	0.36 (0.05–1.5)	0.22
**Charlson Co-morbidity index**	1.15 (0.88–1.49)	0.3
**LEDD**		
*0th-25th percentile*	reference	–
*26th-50th percentile*	1.34 (0.36–5.19)	0.66
*51th-75th percentile*	1.44 (0.40–5.49)	0.58
*76th-100th percentile*	1.33 (0.36–5.2)	0.67
**GI symptom**		
*Dysphagia*	1.44 (0.60–3.56)	0.42
*Nausea*	1.34 (0.46–4.01)	0.59
*Vomiting*	2.02 (0.74–5.57)	0.17
*Epigastric Pain*	1.31 (0.47–3.5)	0.6
*Bloating*	3.59 (1.47–8.88)	**0.005**
*Constipation*	4.24 (0.76–8.01)	0.18

**Fig. 2:**
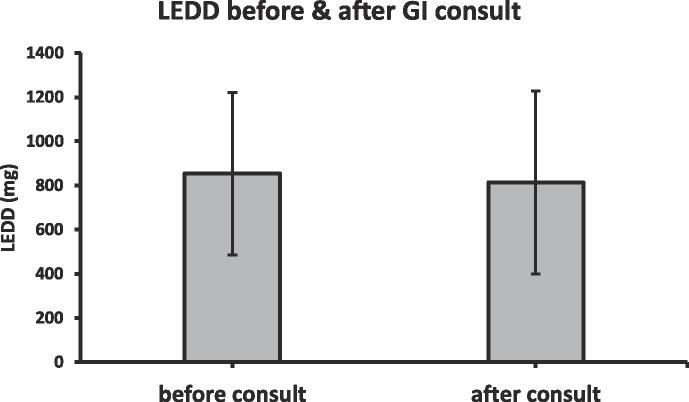
Average Change in LEDD after GI Consult.

Interestingly, dysmotility symptoms did not predict the need for higher LEDD after controlling for age, sex, race, ethnicity, CCI, and UPDRS; evaluation of each symptom with LEDD demonstrated insignificant odds, including with bloating (0.17 < p < 0.99) [Table 3]. GI consultation also tended not to change LEDD, even while controlling for UPDRS motor scores (31.25 mg mean difference; p = 0.75) [Fig. 2].

**Table 3:** Association of dysmotility symptoms with high LEDD.

	Highest quartile LEDD (76th-100th)	
	*Odds ratio (95% CI)†*	*p-value*
**GI symptom**		
*Dysphagia*	1.15 (0.50–2.68)	0.74
*Nausea*	1.51 (0.57–3.99)	0.4
*Vomiting*	1.53 (0.57–4.10)	0.39
*Epigastric Pain*	0.86 (0.30–2.27)	0.77
*Bloating*	1.89 (0.75–4.70)	0.17
*Constipation*	1.00 (0.32–3.69)	0.99

†controlling for age, sex, race, ethnicity, CCI, UPDRS.

Lastly, we found that GI consultation was associated with increased odds of receiving GI-specific treatment and diagnoses. PD patients receiving GI consultation were more likely to receive GI-specific medications (78.2% vs 46.3%; p = 0.001). On sub-group analysis, PD patients receiving GI consultation were more likely to be prescribed nausea/heartburn medications (62.5% vs 25.5%; p < 0.0001) but had comparable rates of laxative prescriptions to those without GI consultation (52% vs 36.6%; p = 0.12) [Table 4.1]. Furthermore, patients who received GI consultation were more likely to receive GI-specific diagnoses of gastroparesis (9.4% vs 0.46%; p < 0.0001) and pelvic floor dysfunction (15.6% vs 0.0% p < 0.0001). No significant difference was noted in a diagnosis of SIBO (3.1% vs. 0.46%, p = 0.24) [Table 4.2].

**Table 4.1:** Association of GI consult with GI medication prescription.

	GI consult, n (%)	no GI consult, n (%)	p-value
**All GI medications‡**	25 (78.2)	100 (46.3)	**0.001**
*Nausea/heartburn medications*	20 (62.5)	55 (25.5)	**<0.0001**
*Laxative medications*	16 (52)	79 (36.6)	0.12

‡omeprazole, pantoprazole, esomeprazole, dulcolax, senna, bisacodyl, metoclopramide, ondansetron, phenergan, famotidine, ranitidine, magnesium citrate, polyethylene glycol, psyllium husk, citrucel, maalox, linaclotide, lubiprostone, TCAs, mirtazapine, prucalopride, linaclotide.

**Table 4.2:** Association of GI consult with GI-specific diagnoses.

	GI consult, n (%)	no GI consult, n (%)	p-value
*Gastroparesis*	3 (9.4)	1 (0.46)	**<0.0001**
*SIBO*	1 (3.1)	1 (0.46)	0.24
*Pelvic floor dysfunction*	5 (15.6%)	0 (0)	**<0.0001**

## 4 Discussion

The lack of association between dysmotility symptoms and LEDD seems to contradict the original presumption that gastric dysmotility affects oral levodopa absorption, as suggested by earlier pharmacokinetic studies [11], [24]. Interestingly, our study is not the first to suggest that gastric dysmotility may have a more limited role in causing motor fluctuations than previously thought. Tanaka *et al.* found no significant difference in delayed gastric emptying (GE) between PD patients with and without motor fluctuations [25], and Nord *et al.* found no relation between delayed GE and levodopa uptake [26]. Possibly, other mechanisms of levodopa malabsorption play a larger role, for example: (1) higher relative abundance of certain bacterial populations like gut bacterial tyrosine decarboxylases can cause premature conversion of levodopa to dopamine [27]. (2) The timing and protein content of foods may cause competitive inhibition of levodopa transport into the bloodstream, also inducing motor fluctuations [28], [29]. In 2012, Heetun *et al.* systemically reviewed existing studies of GE in PD, noting the limitations of each study; in particular, most studies did not control for the protein distribution of meals [15]. (3) Finally, H. pylori infection has been observed in 32–70% of PD patients [30], and eradication of H. pylori in PD patients has been shown to improve levodopa absorption by 21–54% and decrease motor fluctuations [4], [31]. This may explain why levodopa-based gel infusions, which bypass the stomach and main infection site of H. pylori, have shown success in reducing motor fluctuations compared to oral levodopa [32], though the invasive nature of administering gel infusions has limited its practical implementation.

Another alternative etiology of levodopa malabsorption is cross-drug reactions, for example with GI medications. Laxatives used in the treatment of constipation often contain magnesium oxide, which has been linked with in-vitro levodopa degradation [33], [34], [35]. Chronic use of PPIs may likewise contribute to levodopa malabsorption by increasing risk for both gastroparesis and SIBO [36], [37], [38]. Emphasis on the appropriate specialist prescription of GI-medications may therefore be necessary in order to lower the risks of adverse drug-drug interactions and promote better symptom management [39], [40].

While treating symptoms of dysmotility may not ameliorate levodopa-based motor fluctuations as much as prior thought, we found very high prevalence (96.8%) of GI symptoms in PD patients, corroborating the link between dysmotility and PD as suggested by previous studies [16]. Given high prevalence and morbidity of GI symptoms, increased utilization of GI consult for improved management of symptoms may still be advisable.

The association of GI consult with bloating in our study population may reflect regard of bloating as a more difficult-to-treat GI symptom, in addition to bloating being viewed as a proxy symptom for presumed levodopa malabsorption by neurologists. Other GI symptoms were less likely to elicit referral.

Constipation (210/248; 84.7% of patients) and nausea (135/248; 54.4% of patients) were the most prevalent GI symptoms in our cohort. For constipation, wide availability of OTC and non-GI specialist prescriptions for laxatives may lower perceived need for GI consultation when constipation is present as a symptom alone or is of limited severity. With PD-associated nausea, management tends to be more complex, as nausea can be extenuated by anti-PD drugs, and antiemetics that antagonize central dopamine must be avoided. Tailoring antiemetic selection may be better managed with inter-specialty consult, including use of dose-adjusted PPI therapy, dietary management, and additional pharmacotherapy, for example with ondansetron [41], [42] or the peripherally acting dopamine antagonist domperidone [43], [44], which does not cross the blood–brain barrier and has been used as a prokinetic alternative to metoclopramide in PD patients.

Levodopa itself has been reported to cause nausea. Co-administration with carbidopa significantly decreases levodopa-induced nausea by preventing premature conversion of levodopa to dopamine in the bloodstream. When present, levodopa-induced nausea primarily occurs during early levodopa administration [45] in the setting of fast titration regimes up to effective dosages [4], usually abating with continued levodopa treatment [45]. Typically, first response to levodopa-induced nausea is an initial transient reduction in dose before re-titrating levodopa more slowly up to target levels. While any incidence of levodopa-induced nausea in our cohort could have inflated the reported prevalence of GI dysfunction-induced nausea, such inflation is likely minimal. Most patients had already been on levodopa therapy for several years prior to 2018–2022, limiting nausea due to early titration.

Notably, we found that GI consultation was associated with increased odds of receiving GI-specific treatment and diagnoses like gastroparesis and pelvic floor dyssynergia. Such diagnoses were monitored with corresponding treatments, including anti-emetics, procedures like G-POEM, and pelvic floor physical therapy. Though high prevalence rates of SIBO in PD patients are well documented in clinical studies [46], our study population only had 2 documented SIBO cases, limiting statistical power. Non-specificity of symptoms related to SIBO may have limited its diagnosis, as clinical guidelines for breath tests were only established in recent years [47]. SIBO has historically been underdiagnosed in real-word settings, much of which has been attributed to lack of physician awareness regarding SIBO until the past decade.

We note possible limitations of our study, including that the presence of symptoms are relatively imprecise measurements for degree of dysmotility and malabsorption, but were the best available proxy given lack retrospective motility study data. Second, use of LEDD as a measure for changes in motor fluctuations may be limited. Though motor fluctuations are often treated by increasing LEDD, motor fluctuations are also affected by changes in the spacing and frequency of LEDD administered even when overall LEDD dose remains constant.

Our data suggests that dysmotility symptoms and their treatment by GI consult may not affect LEDD. In other words, gastric dysmotility may not influence levodopa-based-motor fluctuations as much as previously presumed. Nonetheless, management of GI symptoms by GI consult remains integral to reduced PD disability rates from non-motor symptom morbidity. Further investigation is warranted, including to understand the larger role of other levodopa malabsorptive mechanisms, specific drug-drug interactions, and more specialized GI treatments. More inter-specialty collaboration and referral would likely prove beneficial.

**Disclosures:** These authors disclose the following: AV has served as a consultant to Alexion Pharmaceuticals, Biogen, and XW Pharma. BK has received research support from NIH, AstraZeneca, Takeda, Gelesis, Medtronic, Genzyme; served as a consultant to Shire, Takeda, Evoke, Phathom and Ironwood; and done educational speaking for Medtronic. The remaining authors disclose no conflicts.

## CRediT authorship contribution statement

**Jocelyn J. Chang:** Conceptualization, Methodology, Investigation, Data curation, Validation, Formal analysis, Visualization, Project administration, Writing – original draft, Writing – review & editing. **Sanjay R.V. Gadi:** Data curation, Writing – review & editing. **Aleksandar Videnovic:** Writing – review & editing. **Braden Kuo:** Supervision, Writing – review & editing. **Trisha S. Pasricha:** Conceptualization, Formal analysis, Supervision, Funding acquisition, Writing – review & editing.

## Declaration of Competing Interest

The authors declare that they have no known competing financial interests or personal relationships that could have appeared to influence the work reported in this paper.
