# Supplementary material for: Impact of outpatient gastroenterology consult on pharmacotherapy and management of gastrointestinal symptoms in Parkinson’s Disease
Source: Clin Park Relat Disord. 2023 Aug 29;9:100215. doi: 10.1016/j.prdoa.2023.100215 (PMC10493246; doi:10.1016/j.prdoa.2023.100215)
Supplement: Supplementary data 1 [file mmc1.docx]

**Supplementary Table 1.**

**GI-specific diagnoses & medications**

| **GI-specific diagnoses** | |
| --- | --- |
| *Gastroparesis*  *SIBO*  *Pelvic floor dysfunction* | |
| **GI medications**  *Nausea/heartburn/abdominal pain* | *Laxatives* |
| Omeprazole  Pantoprazole  Esomeprazole  Ondansetron  Famotidine  Ranitidine  Mirtazapine  *TCAs*  Amitriptyline  Nortriptyline  Desipramine  Imipramine | Dulcolax  Senna  Bisacodyl  Magnesium citrate  Polyethylene glycol  Psyllium husk  Citrucel  Maalox  Linaclotide  Lubiprostone  Prucalopride  Linaclotide |
